# Microplastic pollution in marine ecosystems: sources, impacts, and stakeholder-based solutions

**DOI:** 10.55730/1300-0152.2759

**Published:** 2025-06-23

**Authors:** Seren ACARER ARAT

**Affiliations:** Department of Environmental Engineering, Faculty of Civil Engineering, İstanbul Technical University, İstanbul, Turkiye

**Keywords:** Microplastic, marine, water, marine organisms

## Abstract

Microplastics (MPs) are plastic particles of anthropogenic origin with a size range of 1–5000 μm. Marine MP pollution is increasingly recognized as a significant global environmental challenge. MPs, which enter marine ecosystems from land- and marine-based sources, pose physical, chemical, and biological risks due to the adsorption of various pollutants and the formation of biofilm layers. MPs are found in various organisms from lower to higher trophic levels of the marine food web. The negative effects of MPs on marine organisms depend on their abundance, their characteristics, and the exposure time of organisms to MPs. MPs can adversely affect various vital functions of marine organisms, including survival rate, photosynthesis, growth rate, body composition, reproduction, feeding, and mobility. This review presents the major sources of MPs in the marine environment, as well as their quantities and characteristics. In addition, studies focusing on the potential of MPs to adsorb and transport pollutants, and the levels of accumulation observed in marine organisms, are evaluated. Finally, the responsibilities of stakeholders in mitigating marine MP pollution to maintain marine ecosystem health are discussed. This paper provides a comprehensive review of marine MP pollution, highlighting the main sources of MPs, the pathways through which MPs enter marine ecosystems, the interactions of MPs with coexisting pollutants, their abundance and characteristics in marine organisms, the negative effects of MPs on various marine organisms, and the roles of stakeholders in mitigating MP pollution.

## 1. Introduction

Global plastics production was 1.5 million tons in 1950 (Islam et al., 2023). In The Plastics – The Fast Facts 2024 report published by Plastics Europe, it is stated that global plastics production reached 413.8 million tons in 2023 (Plastics Europe, 2024). Improper disposal of plastics into the environment, low plastic waste recycling rates, the long-term persistence of plastics in the environment, and poor plastic waste management strategies have made plastic and microplastic (MP) pollution a global problem. Plastic pollution in the marine environment has negative effects on aesthetics, the tourism industry, trophic relationships, and biodiversity, leading to negative ecological and socioeconomic consequences (Thushari and Senevirathna, 2020).

MPs (1–5000 μm) are released into the marine environment from different land-based and marine-based sources. MPs coexist with many pollutants in the marine environment and are a good adsorbent for many pollutants due to their small size and large surface area. MPs adsorb many pollutants such as heavy metals, polycyclic aromatic hydrocarbons (PAHs), and pathogens in biofilm layers (Purwiyanto et al., 2020; Deng et al., 2021). They act as vectors, affecting the transport of these pollutants in marine waters and their uptake by marine organisms. Therefore, MPs pose a dual threat to marine ecosystems-both as pollutants themselves and as vectors for adsorbed chemical and biological contaminants. As pollutants they carry chemical and biological risks. It is also worth noting that the combined effects of MPs and the pollutants with which they coexist can lead to more severe negative impacts on marine organisms (Su et al., 2022).

The negative effects of plastics on marine organisms generally include entanglement, choking, internal and external wounds, blockage of the digestive tract, reduced feeding capacity, changes in reproductive capacity, satiation, starvation, changes in vital activities, and decreased quality of life (Gregory, 2009). Studies have reported that MPs of different polymer types, shapes, and sizes are found in marine organisms such as phytoplankton, zooplankton, invertebrates, fish, sea turtles, marine mammals, and seabirds (Acarer Arat, 2024a; Rashid et al., 2025). Studies on phytoplankton indicate that MPs negatively affect cell growth, chlorophyll content, photosynthetic activity, and intracellular metabolic processes, decreasing phytoplankton biomass and biodiversity—the primary producers in marine ecosystems (Casabianca et al., 2021; Dong et al., 2022; Cousins et al., 2024). MPs can be transferred to marine organisms not only through direct ingestion, but also indirectly by consuming organisms that have previously ingested MPs. Exposure of zooplankton to MPs negatively affects their survival, algal nutrition, growth, and reproductive success (Cole et al., 2013; Lee et al., 2013; Kosore et al., 2018; Isinibilir et al., 2020). Studies have shown that MPs accumulate in the kidneys, liver, spleen, muscles, fat, stomach, intestines, brain, and reproductive organs of higher trophic level organisms such as fish, sea turtles, marine mammals, and seabirds (Atamanalp et al., 2021; Wootton et al., 2021; Navarro et al., 2023; Courville et al., 2024; Eryaşar et al., 2024; Costello et al., 2025). MPs negatively affect survival rate, mass, head/body ratio, swimming behavior, mobility, food utilization efficiency, and the immune system in fish (Barboza et al., 2018; Mahmood et al., 2024; Rashid et al., 2024a; Rashid et al., 2024b). MPs, which particularly accumulate in the reproductive system of sea turtles (Costello et al., 2025), also pose a potential threat to their reproductive success. As for seabirds, the presence of different types of MPs in their gastrointestinal tract and fecal samples has also been confirmed (Navarro et al., 2023; Ammendolia et al., 2025). The false sense of satiety caused by plastic fragments in seabirds reduces food intake and leads to energy deficiency, while also causing internal organ damage that may result in severe health complications or even death (Acarer Arat, 2024a; Rashid et al., 2025). MPs adversely affect the vital functions of all organisms, from low trophic level phytoplankton to high trophic level marine organisms, such as energy production, survival, and reproduction. The adverse effects of microplastics on individual organisms can seriously threaten the health, biodiversity, and sustainability of marine ecosystems, posing a long-term risk to broader populations and disrupting ecosystem functioning (Aminah et al., 2023). Moreover, since MPs accumulate in various tissues of marine organisms consumed by humans, they may ultimately reach humans and pose a potential risk to human health.

This paper presents (1) the land-based and marine-based sources of MPs in marine ecosystem, (2) the pollutants adsorbed onto their surfaces and their role as vectors in marine waters, (3) their abundance and physicochemical characteristics in the marine environment, (4) the characteristics of MPs accumulated in marine organisms across different trophic levels along with their adverse biological effects, and (5) stakeholder-based mitigation strategies targeting manufacturers, consumers, policymakers, and researchers to prevent MP pollution in marine ecosystems. Addressing MP pollution in the marine environment through a holistic framework—from identifying sources and characterizing their properties to evaluating biological accumulation and proposing stakeholder-based mitigation strategies—provides a comprehensive basis for effectively managing and preventing MP pollution in marine ecosystems.

## 2. Sources of microplastics in the marine ecosystem

**Figure 1:**
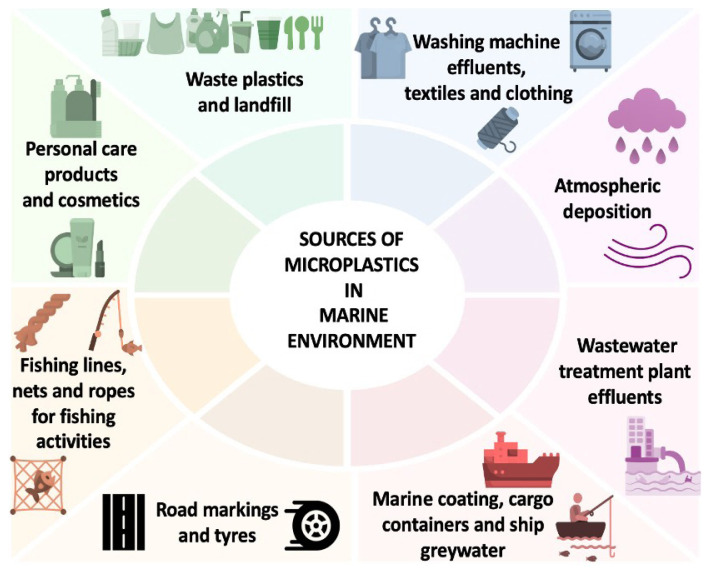
Sources of MPs in the marine environment.

MPs are plastic particles between 1 μm and 5000 μm in size (Acarer Arat, 2024b). Primary MPs are small-sized MPs that are intentionally produced to impart various properties to products or to produce large-sized plastic products. Examples of primary MPs include virgin polymeric microbeads used to modify the properties and improve the performance of personal care products such as facial cleansers, shower gels, exfoliating scrubs, toothpaste, as well as cosmetic products such as nail polish and eye shadow (Tsiota et al., 2018). Small-sized pellets used in the production process of plastic products are another example of primary MPs. Secondary MPs are formed when large-sized plastics are fragmented by physical, chemical, or biological factors alone or in combination. The presence of MPs in the marine environment, rivers, tap water, bottled drinking water, wastewater, landfill soils, landfill leachate, agricultural soils, air, food, and humans has been reported in studies (Acarer, 2023a; Acarer Arat, 2024b). Figure 1 shows the sources of MPs in the marine environment. Approximately half of all plastic has accumulated in landfills or been dumped in the wild since 1950 (d’Ambrières, 2019). Physical, chemical, and biological factors lead to the formation of secondary MPs through the breakdown of plastic waste in areas where plastic waste is stored (Acarer Arat, 2024b). MPs are found in landfill soils in different polymer types such as polyethylene terephthalate (PET), polyvinyl chloride (PVC), cellulose acetate (CA), polymethyl methacrylate (PMMA), polyamide (PA), polyethylene (PE), polystyrene (PS), and polypropylene (PP), and in different forms such as fiber, fragment, film, sphere, pellet, and foam (Mahdavi Soltani et al., 2022; Mahesh et al., 2023; Pratiwi et al., 2024). The existence of MPs of different polymeric types such as PE, PP, PS, PVC, PET, PA, PMMA, polyurethane (PU), acrylonitrile butadiene styrene (ABS) resin, ethylene vinyl acetate (EVA), epoxy resin (EP), phenol-formaldehyde resin (PF), alkyd (ALK), polydimethylsiloxane (PDMS), and polytetrafluoroethylene (PTFE) has been reported in leachate generated in solid waste landfills (He et al., 2019). MPs in landfills can end up in the marine environment by being carried by environmental factors such as surface runoff and wind (Pourebrahimi and Pirooz, 2023). Even if the landfill leachate is treated in a leachate treatment plant where membrane technologies that provide superior purification are applied, MPs are not completely removed from landfill leachate (Kara et al., 2023). Therefore, MPs can also end up in the marine environment through the discharge of treated landfill leachate.

Surface runoff plays a significant role in transporting not only MPs from landfills but also MPs from urban and highway sources into the marine environment. Road runoff includes road markings, tire wear particles, vehicle paints, exterior paints, and artificial grass. These are washed away by rainwater and usually enter the water environment directly in rural areas, while in urban areas they enter separate sewers or combined sewers (Wang et al., 2019). Road marking contains a variety of polymers including ALK, polyester (PEST), acrylic (ACR), and epoxy (EP) (Burghardt et al., 2022). Vehicle tires are complex structures consisting of components such as synthetic rubber, natural rubber, PEST, nylon, pigments, carbon black, and chemical additives (Siddiqui et al., 2022). MP pollution from tires can pose a significant threat to rivers and marine ecosystems, especially near busy roads. It has been estimated that plastics formed from tire wear and tear contribute to approximately 5%–10% of the global amount of plastic reaching the oceans (Kole et al., 2017).

MPs used in personal care and cosmetic products mix with water during users’ daily activities, enter the domestic wastewater system, and are transported to wastewater treatment plants via sewage systems. However, the existing primary, secondary, and tertiary treatment processes in wastewater treatment plants cannot completely remove MPs from wastewater (Acarer, 2023b). Thus, MPs originating from personal care products, cosmetics, and other plastics are released into the aquatic environment through the discharge of wastewater treatment plant effluent. A study showed that MPs of PE, low-density PE (LDPE), and ethylene-propylene polymer types, with round and irregular shapes, sizes ranging from 238.55 ± 50.74 μm to 450.69 ± 174.9 μm, and white or transparent colors, are present in skin creams, shaving creams, baby creams, face washes, and face scrubs (Gamage and Mahagamage, 2024). The study showed that face scrubs and face washes emitted 36 ± 0.2 g and 0.2 ± 0.05 g of MPs per product, respectively (Gamage and Mahagamage, 2024).

Microfibers in washing machine effluents originating from the washing of synthetic textiles are the main source of fibers in domestic wastewater. The abundance of microfibers in washing machine effluents varies depending on the load of the clothes in the washing machine, the type of fabric, the age of the fabric, the washing temperature, the detergent properties, and the washing process (Le et al., 2022). MPs of different polymer types, such as PET, PE, PP, PS, PA, PU, and polyacrylonitrile (PAN), are found in washing machine wastewater (Luu et al., 2025; Park et al., 2025). Many studies have shown that fiber-shaped MPs dominate the influent of wastewater treatment plants (Acarer, 2023b). The abundance of fibers in the influent of wastewater treatment plants is mainly associated with the domestic laundry process. MPs in domestic wastewater and industrial wastewater (Haque et al., 2022) are significantly retained in wastewater treatment plants through preliminary, primary, secondary, and tertiary treatment (Acarer, 2023b). Industrial wastewater and effluents from industrial wastewater treatment plants are potential sources of MPs. A study by Wang et al. (2020a) showed that the abundance of MPs in wastewater collected from chemical, electroplating, and machinery manufacturing industrial plants in Changzhou, China, ranged from 8–36 particles/L, and PE-type MPs were found in all wastewaters from these industries (Wang et al., 2020a). The same study showed that the abundance of MPs in the effluent of an industrial wastewater treatment plant treating wastewater from the textile, machinery manufacturing, chemical, and steel industries ranged from 6–12 particles/L, and MPs of PP and PS types were found in all effluents (Wang et al., 2020a). Another study by Haque et al. (2022) revealed the presence of MPs in all wastewaters from dyeing, washing, printing, pharmaceutical, and battery industries. The study showed that the highest MP abundance was in dyeing industry wastewater (431 ± 187 items/L), and the lowest was in battery industry wastewater (7 ± 3 items/L). Many studies have shown that wastewater treatment plants remove MPs with high efficiency (Acarer, 2023b). However, when the amount of water discharged by wastewater treatment plants (WWTPs) and the number of WWTPs are considered, it becomes evident that billions of MPs are released into the receiving environment each day (Jiang et al., 2022; Akdemir and Gedik, 2023). Studies have shown that MPs of different polymer types, such as PE, PP, PS, PET, PA, PVC, ABS, polyvinyl alcohol (PVA), EVA, and rayon, are found in the effluents of WWTPs (Long et al., 2019; Jiang et al., 2022; Van Do et al., 2022).

Rivers play an important role in transporting plastics into the marine environment. In a study conducted by Terzi et al. (2025), it was reported that the abundance of MPs in water samples collected from 29 river mouths discharging into the Black Sea along the southern Black Sea coast of Türkiye varied between 1.03–29.8 particles/m^3^, and the average MP abundance was 9.63 ± 1.27 particles/m^3^. In the study, different polymeric types of MPs, such as PE, PP, and PA, were detected at almost all sampling points except one, with PET-type MPs being dominant. Fiber-shaped MPs were dominant in water samples collected from river mouths, and the presence of fragment-, film-, and sphere-shaped MPs was also determined (Terzi et al., 2025).

Although land-based MPs are the main source of MP pollution in the marine environment, marine-based MPs also contribute significantly to this pollution. Fishing, shipping, cargo damage accidents, and aquaculture are among the marine-based sources of MPs. Fishing gear fibers, nets, and ropes are usually made of polymers such as PEST, PE, PP, and PA (Corniuk et al., 2023; Karadurmuş and Bilgili, 2024). A study showed that fishing ropes and nets have a higher MP emission potential than fishing lines (Wright et al., 2021). Abandoned plastic fishing gear releases MPs into the marine environment even in benthic conditions where photodegradation is limited. For instance, a study by Welden and Cowie (2017) showed that the mass of 10 mm diameter PE, PP, and nylon ropes placed on the seabed at a depth of 10 m decreased by an average of 0.39%, 1.02%, and 0.45% per month, respectively. The mechanical hauling of ropes in the marine environment also produces large amounts of MPs. Napper et al. (2022) reported that pulling 50 m of < 2-year-old and ≥ 2-year-old ropes from a boat could emit 700–2000 and 36,000–38,000 MPs, respectively. The study by Napper et al. (2022) revealed that, considering 4500 active fishing vessels spending 100 active days at sea in the UK, this could result in between 326 million and 17 billion MPs entering the ocean per year.

MPs from shipping activities are released from coating layers, waste, and greywater of ships. Paints are used on ships and boats due to their polymer backbones (PEST, PU, PS, EP, ALK, polyacrylates, and alkyls), which act as binding agents (Soon et al., 2024). Factors contributing to the release of MPs into the marine environment include paint damage, abrasion, underwater cleaning, sanding, and rinsing of tools used in the painting process (Soon et al., 2024). Considering the similarity of activities on ships to domestic ones, the entry of MPs into the marine environment is inevitable due to the discharge of greywater from ships into the receiving environment. For instance, a study by Jang et al. (2024) showed that the abundance of MPs in the laundry, cabin, and galley greywater of a vessel was 177,667, 133,833, and 75,000 items/m^3^, respectively. The dominant MP shape, polymer type, and size were fiber (66%), PEST (53%), and 100–200 μm, respectively. In the study, considering the greywater production rate of 0.15 m^3^/person·day during navigation, the annual MP emission per person was calculated as 4.1 × 10^6^ items/person·year (Jang et al., 2024).

Although the irregular morphology, wide size range, and diverse chemical composition of MPs prevent a complete understanding of their migration mechanisms in the atmosphere (Chen et al., 2023), it is well known that atmospheric MPs can be transported hundreds to thousands of kilometers from their source (Allen et al., 2022). Atmospheric MPs make a nonnegligible contribution to MP pollution in the marine environment. Ding et al. (2021) collected samples of atmospheric MPs from the northwestern South China Sea and reported that the dry deposition of MPs to the marine environment in the fall was 1.4 × 10^3^ tons, leading to a larger contribution than riverine inputs in this region.

## 3. Quantitative and qualitative assessment of marine microplastics

The lack of a globally standardized method for MP analysis results in methodological differences in research. Currently, sampling tools with different mesh sizes, chemical components, and chemical concentrations are used to determine the abundance of MPs in water samples. MP abundance in marine water and marine sediments is reported in different volume-based or surface area-based units (e.g., particles/L, particles/m^3^, particles/kg, particles/m^2^, particles/km^2^) depending on the sampling method. Methodological differences in MP sampling and analysis make it difficult to compare studies conducted in the same or different marine environments. This prevents accurate mapping of the worldwide distribution of MP pollution and makes it difficult to assess differences in the temporal and spatial distribution of MP pollution in the marine environment.

To isolate MPs by digesting organic matter in a marine water sample, the chemical used in the digestion process should not affect the structure, volume, weight, size, color, or shape of the MPs, as this can lead to an inaccurate estimate of their abundance and characteristics (Pfeiffer and Fischer, 2020). The use of different mesh sizes in studies investigating MP pollution in the marine environment directly affects the size distribution of MPs captured. The type and proportion of chemicals used in the pretreatment of the sample (e.g., removal of organic matter, density separation) before microscopic imaging of MPs may lead to underestimation or overestimation of MP abundance in the sample. For instance, sodium chloride (NaCl) (density: 1.2 g/cm^3^) is a widely used chemical in the extraction of MPs in samples collected from marine environments using the density separation method (Aksu et al., 2023). However, while NaCl is effective for the flotation and transfer of MPs with lower density (e.g., PE, PP) to the supernatant, it may not efficiently separate MPs with higher density (e.g., PET, PVC, PA, PC, PU), potentially leading to an underestimation of their true abundance in marine water samples.

Table summarizes representative studies reporting the abundance, shapes, colors, and polymer types of MPs in open oceans, enclosed seas, and bays from different regions of the world. The accumulation of MPs in the marine environment is a complex process that depends not on a single factor but on multiple factors. The factors affecting the abundance of MPs in the marine environment are not easily understood because many processes—such as the intensity and types of human activities, hydrodynamic processes (e.g., waves, currents), and atmospheric transport—contribute to their accumulation. A recent study by Hakim et al. (2023) showed that the abundance of MPs detected in sand (291–457 particles/m^2^), sediment (627 particles/100 g), and water (327–624 particles) at a pristine beach was similar to that found in sand, sediment, and water samples from beaches serving multiple functions such as markets, tourism, and harbors. Another study by Jiang et al. (2020) revealed that the abundance of MPs in marine water samples collected from 16 different points of the Yellow Sea in January, April, and August varied between 4.5 ± 1.8 – 6.5 ± 2.1 items/L. The researchers reported that MPs were transported to the marine environment due to intense air pollution in the dry season (January) and surface runoff in the wet season (August) and that hydrodynamic processes were a factor affecting the abundance of MPs (Jiang et al., 2020).

MP pollution in the marine environment varies both spatially and temporally. In a study in which the abundance of MPs in seawater samples collected from four sites in Sanya Bay ranged from 15.50–22.14 items/L, MP abundance was found to be relatively higher near the Sanya River estuary, which is heavily affected by human activities, compared to the open-water site under military control (Lei et al., 2021). A study by Aksu et al. (2023) showed that the highest abundance of MPs in seawater samples collected from 10 different points of İzmir Bay during the wet season occurred in regions characterized by marina operations, passenger piers, shipyard activities, stream discharge, anticyclonic circulation, and WWTP discharge. The same study revealed that the abundance of MPs was highest in seawater samples in the dry season, especially in points close to the passenger piers where passenger and vehicle transportation is carried out (Aksu et al., 2023). Another study has shown that MP concentrations are 4–27 times higher in sites nearshore in the northeastern Pacific Ocean than in sites offshore (Desforges et al., 2014). The abundance of MPs in marine water varies not only by location but also by the depth at which the water sample is collected. The study conducted by Öztekin et al. (2024) at seven sampling points in Hamsilos Bay, an important natural protected area for tourism and fishing on the Sinop coast of the Black Sea, revealed that the abundance of MPs varied between 5.58 ± 6.12 – 8.12 ± 9.17 particles/m^3^. The study conducted at five different depths at seven stations showed that MP abundance decreased with increasing depth (Öztekin et al., 2024).

Considering the studies conducted to date, it is evident that a standardized MP analysis method needs to be developed and implemented globally to better understand MP pollution in the marine environment and to develop effective strategies to mitigate it. Further research on the abundance of MPs in marine environments at different times and locations, conducted using a standardized method, may contribute to more accurate mapping of the global distribution of MP pollution, monitoring of its temporal changes, and the development of effective strategies to reduce it.

MP fragments in the marine environment are generally secondary MPs formed by the breakdown of large-sized hard plastics such as bottles, caps, and containers over time due to exposure to environmental factors (Uaciquete et al., 2024). Fiber-shaped MPs in the marine environment mainly originate from wastewater treatment plant effluents, fishing nets, ropes, and clothing (Desforges et al., 2014; Georgieva et al., 2023). The polymer type of MPs in the marine environment does not necessarily indicate their origin. On the other hand, the polymer type of MPs, when considered together with other factors such as shape, provides an important clue in predicting the possible origin of MPs. The presence of MPs composed of various polymer types—such as PE, PP, PS, PEST, PET, PVC, PA, PU, PAN, PC, ABS, polyacrylic acid (PAA), cellophane (CP), styrene-butadiene (SBR), polybutadiene (PB), and tetrafluoroethylene-hexafluoropropylene (FEP)—has been reported in marine waters.

As a remarkable result, PE was reported to be the dominant polymer type in MPs in marine waters in most parts of the world, such as the Bohai Sea (Zhang et al., 2017), eastern Aegean Sea (Aksu et al. 2023), western Mediterranean Sea (Baini et al., 2018), eastern Mediterranean Sea (Adamopoulou et al., 2021), Yellow Sea (Jiang et al., 2020), western Arctic Ocean (Ikenoue et al., 2023), and the seas in Türkiye (Aydın et al., 2023). Of the 413.8 million metric tons of plastic produced worldwide in 2023, 26.2% was PE, making it the most widely produced plastic type globally (Plastics Europe, 2024). The prevalence of PP as well as PE MPs in marine waters is not surprising, as PP also has a significant production percentage worldwide (Plastics Europe, 2024) (Baini et al., 2018; Adamopoulou et al., 2021; Lei et al., 2021; Takarina et al., 2022; Aksu et al., 2023; Hakim et al., 2023; Ikenoue et al., 2023; Uaciquete et al., 2024) PE and PP are widely used in everyday life in plastic bags, packaging, toys, and bottle caps. Another reason PE and PP are commonly found in marine waters is that the density of both polymers is lower than that of seawater, making them easier to sample (Takarina et al., 2022). Fragments of PE, PP, PS, and PVC are usually formed by the breakdown of materials such as cups, bottles, and plastic bags into smaller pieces. Scrubbers made of ACR and PEST are commonly used in air blasting to remove paint and rust from boat hulls and machinery, and they can enter the marine environment (Cole et al., 2011). PEST and PA fibers often originate from the washing of synthetic textiles and enter the marine environment directly or through the discharge of wastewater treatment plant effluents (Acarer, 2023b). Polymers such as PE, PP, PEST, PA, and PVA, used in fishing gear, also release fiber-shaped MPs into the marine environment (Öztekin et al., 2024).

MPs are found in a variety of colors in the marine environment, including white, black, transparent, grey, blue, red, pink, purple, yellow, green, brown, and multicolored forms. Studies have shown that white (Zhang et al., 2017; Baini et al., 2018; Zhang et al., 2020; Aksu et al., 2023), black (Lei et al., 2021; Hakim et al., 2023), and blue MPs (Desforges et al., 2014; Kanhai et al., 2017; Emberson-Marl et al., 2023) are the most detected in marine environments. Color can be a parameter that helps to identify possible sources of MPs. White MPs originate from packaging, covering materials used in greenhouse cultivation, paints used in marine coatings, textiles, and personal care products. Blue MPs originate from blue plastic waste such as plastic bottle caps, industrial processes, clothing, ship coatings, and ropes. Black MP sources include worn tires, textiles, industrial processes, ropes, and plastic items (e.g., plastic bags, cups, food storage containers). The rate of photodegradation and the degree of photooxidation vary according to the color of the MPs (Li et al., 2024b). Different color wavelengths of MPs affect photoaging by affecting solar absorbance and UV transmittance (Zhao et al., 2022). Color is also an important factor in the interaction of MPs with living organisms. For instance, some fish use color vision to recognize and selectively ingest MPs, and color preference may vary among fish species (Horie et al., 2024). A study was conducted to investigate the ingestion of PE fragments of five different colors (white, black, blue, green, and red) by goldfish (*Carassius auratus*) at a concentration of 100 items/L with 0.3 g of fish feed (Xiong et al., 2019). The results of the study indicated that goldfish ingested more green and black MPs than white, red, and blue (Xiong et al., 2019). Future studies should continue to investigate the color of MPs because color is an important factor for identifying the possible source of MPs and understanding the biological interactions of MPs.

## 4. Impacts of microplastics on marine ecosystems

### 4.1. Microplastics as vectors of marine pollutants

MPs are good adsorbents for environmental pollutants due to their small size, high specific surface area, hydrophobicity, and porous structure, which contribute to increased adsorption ability. MPs can adsorb many pollutants, including polycyclic aromatic hydrocarbons (PAHs), polychlorinated biphenyls (PCBs), heavy metals, pharmaceuticals, pesticides, natural organic matter, and disinfection by-products (Purwiyanto et al., 2020; Li et al., 2024c). The mechanisms that are effective in the adsorption of pollutants onto MPs include electrostatic interactions, hydrophobic interactions, noncovalent interactions, and partitioning (Fu et al., 2021). The high surface areas and hydrophobicity of MPs make them good carriers for microbial colonization. Biofilm formation on plastic surfaces involves the attachment of microorganisms, the release of extracellular polymeric substances (EPS) (e.g., polysaccharides, proteins, lipids), and the proliferation of microorganisms in biofilms. Bacteria, fungi, and algae are commonly found in biofilms (Sharma et al., 2023). This indicates that MPs are not only a physical pollutant but also act as a carrier of many microorganisms. A study has shown that potential opportunistic pathogens, including *Vibrio*, *Enterobacteriaceae*, and *Tenacibaculum*, are found in the biofilm of MPs (Deng et al. 2021). In addition, the EPS matrix makes the MP sticky during biofilm formation, which facilitates the adsorption of pollutants from the marine environment (Rummel et al., 2017; Deng et al., 2021). Biofilms of MPs in freshwater and MPs in marine water differ because freshwater microorganisms and seawater microorganisms are adapted to different pH values (Sharma et al., 2023). Biofilm formation increases the density of MPs and reduces the buoyancy of MPs (Rummel et al., 2017). This may lead to MPs migrating to deeper waters and accumulating on the seafloor. Thus, the formation of biofilms on MPs can lead to several phenomena such as altered vertical distribution of MPs in the marine environment, accumulation of MPs in marine sediments, and increased adverse effects of MPs on deep water organisms.

The physical and chemical properties of MPs play an effective role in the adsorption of pollutants on MPs. For instance, a study by Mamtimin et al. (2023) revealed that the adsorption capacities of PS MPs with −COOH functional groups at three different sizes (82 nm, 200 nm, and 500 nm) were 0.57 mg/g, 0.25 mg/g, and 0.20 mg/g for As^3+^, and 0.37 mg/g, 0.32 mg/g, and 0.21 mg/g for As^5+^, respectively. The study showed that the adsorption capacities of PS with −NH_2_ functional groups at 80 nm size were 0.41 mg/g and 0.27 mg/g for As^3+^ and As^5+^, respectively. The study revealed that MPs with smaller sizes and oxygen-containing functional groups (−COOH) have higher As adsorption ability (Mamtimin et al., 2023). In addition to the physical and chemical properties of MPs, environmental factors such as pH, ionic strength, and the concentration of other pollutants play a significant role in the adsorption process of different pollutants onto MPs. In the study by Wang et al. (2022), three different antibiotics—amoxicillin, ciprofloxacin, and tetracycline—were adsorbed onto PE, PP, PS, and PVC MPs, and it was determined that the adsorption process was governed by physical (electrostatic interaction, hydrophobicity, crystallinity, surface area, particle size, diffusion) and/or chemical mechanisms (ion exchange, electron sharing, and π-π conjugation). The study showed that the adsorption of antibiotics onto MPs tends to decrease with increasing ionic strength, that adsorption capacity decreases at pH values above 5, and that antibiotic adsorption may increase or decrease depending on the type and concentration of organic matter in the medium (Wang et al., 2022).

Aging of MPs by environmental factors (e.g., UV radiation, heat, physical abrasion, chemical oxidation, and biological processes) causes changes in their adsorption capacity and mechanisms by altering surface morphology, functional groups, hydrophobicity, and crystallinity (Hu et al., 2024). Aging processes increase the oxygen content and surface roughness of MPs, enhancing their adsorption capacity for pollutants. The study conducted by Gao et al. (2021) revealed that PS, PVC, PA, and PET MPs in seawater, aged for 3 months under UV irradiation at 340 nm using a 20 W lamp in an aging chamber, developed pores, cracks, and folds on their surfaces. The study reported that the surface roughness of MPs aged in natural seawater under UV irradiation was higher than that of MPs aged in air, possibly due to the presence of various microorganisms in seawater promoting degradation. The study also reported that aged PVC and PS had higher Cu^2+^ and Cd^2+^ adsorption capacities in seawater compared to their unaged counterparts (Gao et al., 2021).

In another study by Hu et al. (2024), PA and polyactic acid (PLA) MPs were aged for 240 h using a 500 W mercury lamp at 200–450 nm wavelength to mimic photoaging in nature. The study showed that the color of MPs changed from white to yellow as the aging process progressed, indicating that the MPs underwent UV-induced photooxidation and that their smooth, dense surfaces transformed into rough, pitted, cracked, and wrinkled surfaces. The study showed that aged PA and PLA MPs adsorbed more Cu^2+^ than their virgin counterparts at pH values ranging from 1 to 7 and salinity levels ranging from 0% to 3.5%. It was determined that at pH > 5, Cu^2−^ precipitated, reducing its availability for adsorption. At pH < 5, the high H^+^ concentration and electrostatic repulsion hindered the adsorption process. Similarly, the adsorption ability of MPs for Cu^2+^ decreased as salinity increased due to competition from Na^+^ with free Cu^2+^ for adsorption sites on the MP surface (Hu et al., 2024).

As a result, MPs are not passive pollutants in the marine environment due to their high adsorption capacity, but dynamic vectors of pollution that include chemicals and microorganism colonies. The physicochemical properties of MPs, aging processes, and environmental conditions affect the adsorption process and the amount of pollutants adsorbed on MPs. Acting as vectors for pollutants, toxic MPs increase the risk of pollutant bioaccumulation along the marine food chain, negatively affecting both the food chain and energy flows.

### 4.2. Microplastic pollution and its effects on marine trophic levels

#### 4.2.1. Microplastic pollution and its effects on lower trophic levels

The effects of MPs on phytoplankton vary depending on the polymer type of MPs (Su et al., 2022), the concentration of MPs (Wang et al., 2020b; Su et al., 2022), and the exposure time to MPs (Guo et al. 2020; Wang et al. 2020b). MPs have many negative effects on marine phytoplankton, such as promoting hetero-aggregate formation and affecting cell growth, photosynthesis, and chlorophyll-a content (Casabianca et al., 2021). MPs inhibit phytoplankton growth due to reduced light penetration, aggregation, and toxicity (Cousins et al. 2024). A study showed that a 4-day short-term exposure of *Phaeodactylum tricornutum* to PE and PVC MPs at concentrations ranging from 0.05–50 g/L did not result in significant changes in algal growth and lipid concentration per cell, while a 9-day long-term exposure decreased algal growth and induced lipid accumulation (Guo et al., 2020). It has been reported that phytoplankton biomass and diversity will decrease by 2100 under predicted temperature conditions (Cousins et al., 2024). Besides the effect on phytoplankton growth, other negative effects of MPs include the reduction of chlorophyll content and the inhibition of photosynthetic activity. For instance, a study investigating the effects of 1 μm PVC MPs on diatoms (*Phaeodactylum tricornutum*, *Chaetoceros gracilis*, and *Thalassiosira* sp.) showed that all algal species exhibited a decrease in cell density and chlorophyll content at PVC concentrations between 25–200 mg/L (Wang et al., 2020b). Another study revealed that the growth (minimum EC_50_ value of 91.75 mg/L) and photosynthetic activity of the marine diatom (*Phaeodactylum tricornutum*) were negatively affected by exposure to PMMA MPs at different salinities ranging from 25‰–45‰ for 10 days (Dong et al., 2022). MPs and various pollutants coexist in the marine environment, and MPs act as adsorbents for these pollutants. The combined effect of MPs and pollutants on marine organisms is higher than the effect of MPs alone. One study evaluated the effects of PET and PS MPs and one of the PAHs, benzo[a]pyrene, both individually and in combination on the growth of *Chaetoceros muelleri*. After 15 days of cultivation, the combined effect of 200 mg/L PS MPs + 150 μg/L benzo[a]pyrene on the inhibition rate of *C. muelleri* (55.18%) was higher than that of 150 μg/L benzo[a]pyrene alone (inhibition rate [IR] = 40.34%) and 200 mg/L PS MPs alone (IR = 1.26%) (Su et al., 2022). Similarly, the combined effect of 200 mg/L PET MPs + 150 μg/L benzo[a]pyrene on the inhibition rate of *C. muelleri* (52.66%) was higher than that of 150 μg/L benzo[a]pyrene alone (IR = 40.34%) and 200 mg/L PET MPs alone (IR = 4.36%) (Su et al., 2022).

Zooplankton occupy a trophic position between phytoplankton and higher trophic levels, and serve as important food sources for secondary consumers. Small animals that spend their entire life as plankton (such as copepods) belong to the holoplankton category, while animals that spend only part of their life cycle as plankton (such as sponges, anemones, benthic larvae, seaweed spores, fish larvae) belong to the meroplankton category (Dipper, 2022). Zooplankton at lower trophic levels of the marine food chain ingest MPs as food (Desforges et al., 2015). Previous studies have reported that zooplankton can ingest MPs of various shapes, polymer types and sizes (Desforges et al., 2015). Thus, MPs can be transferred between trophic levels through ingestion by organisms at lower trophic levels of the marine food chain, such as zooplankton, as well as by organisms at higher trophic levels. A study, in which zooplankton sampling was carried out in the western English Channel followed by exposure tests using commercial fluorescent PS spheres (1.7–30.6 μm), showed that 13 zooplankton taxa were capable of ingesting PS spheres and that copepods, decapod larvae, and bivalve larvae were among the organisms that ingested the MPs (Cole et al., 2013). The number of MPs ingested by zooplankton is positively correlated with the abundance of MPs in the marine environment, meaning that as MP abundance increases, both the exposure of zooplankton to MP pollution and their capacity to ingest MPs may also increase (Desforges et al., 2015).

The characteristics of MPs (e.g., shape, size, color, polymer type) and the feeding habits of zooplankton also affect the uptake of MPs by zooplankton. For instance, Kosore et al. (2018) investigated MP pollution in seawater samples collected from 11 stations along the Kenyan coast and MP ingestion by zooplankton samples (Chaetognatha, Copepoda, Amphipoda, and fish larvae) collected from the stations. The study showed that the abundance of MPs in surface seawater averaged 110 items/m^3^, and the dominant shape, color, and polymer type of MPs in seawater were filament, white, and PP, respectively. The study revealed that Chaetognatha, Copepoda, Amphipoda, and fish larvae ingested 0.46, 0.33, 0.22, and 0.16 particles per ind, respectively, and that the ingested MPs were in the form of filaments and fragments, of LDPE polymer type, 0.01–1.6 mm in size, and mainly black. The study emphasized that filaments, which predominate in seawater and can reduce their apparent size for ingestion by organisms due to their self-bending and folding properties, are more readily ingested by zooplankton. Moreover, the study concluded that copepods feeding near the surface have a high ability to ingest LDPE MPs due to their tendency to ingest PE-type MPs, which have lower density than seawater (Kosore et al., 2018). Another study by Botterell et al. (2020) showed that although PS beads, nylon fibers, and nylon fragments were all ingested by copepods (*Calanus helgolandicus* and *A. Tonsa*) and *Homarus gammarus* larvae, each species selectively preferred one shape over the others. Tests with *Calanus helgolandicus* showed that there were differences in the ingestion rates of different shapes of MPs (beads, fibers, fragments), and that from the highest to the lowest ingestion rate, the shapes of MPs were fragments (500.3 ± 83.9 fragments/copepod·h), fibers (251.9 ± 134 fibers/copepod·h), and beads (144.5 ± 38.4 beads/copepod·h), respectively (Botterell et al., 2020).

It is also worth noting that investigating the uptake of MPs by zooplankton and other marine organisms after environmental exposure is critical to understanding the ecological impacts of MP pollution. Commercial MPs are often studied in the laboratory in their virgin form, which may not reflect the processes and interactions that MPs undergo in the environment. MPs undergo significant changes in their physical and chemical properties in the environment through aging processes and biofilm formation. These changes can significantly affect the interactions of these particles with biotic elements, especially organisms such as zooplankton. For instance, compared to virgin PS MPs, aged PS MPs kept in filtered seawater in the dark for 3 weeks to allow biofilm formation were found to be ingested at higher rates by more individuals in two copepod species (*C. finmarchicus* and *A. longiremis*) (Vroom et al., 2017).

Exposure of zooplankton to MPs leads to many negative consequences such as reduced survival, increased mortality, slowed growth, reduced reproduction, decrease in algal feeding rate, early settlement of larvae, and significant changes in the size spectrum of algal prey (Cole et al., 2013; Lee et al., 2013; Y. Gao et al., 2023). Consistent with these adverse effects, findings from a study in Türkiye showed how rapidly MPs can disrupt vital parameters of zooplankton (Isinibilir et al., 2020). The study conducted in Türkiye revealed that exposure to PS MPs led to a decline in the vital physiological parameters of the copepod *Calanus helgolandicus* from the Marmara Sea as early as the first day, likely as a result of accelerated starvation and/or the traumatic or toxic effects induced by the MPs (Isinibilir et al., 2020). This leads to the decline of zooplankton populations and their inability to fulfill their role in the marine ecosystem. MP pollution in marine ecosystems not only harms zooplankton but also has far-reaching consequences by affecting their ecological functions. MP-ingesting zooplankton grazing on primary producers can be reduced, leading to significant changes in biogeochemical rates related to dissolved oxygen in seawater. This indicates that MP pollution could reach levels that potentially affect the global deoxygenation trend (Kvale et al., 2021).

#### 4.2.2 Microplastic pollution and its effects on higher trophic levels

Ingestion of MPs by marine organisms at higher trophic levels occurs through direct and accidental ingestion (primary ingestion) as well as through predation by prey that has previously consumed MPs (secondary ingestion) (Wootton et al., 2021). The presence of MPs of different shapes, colors, and polymer types in fish has been reported in many studies. The study conducted along the coast of Türkiye on the Black Sea demonstrated the presence of 168 and 264 MPs in red mullet (*Mullus barbatus*) (n = 82) and pontic shad (*Alosa immaculata*) (n = 82), respectively. MP abundance was identified across various tissues, including the gastrointestinal system, gills, muscle, and brain. Three main types of MPs were fibers (51%), fragments (7%), and pellets (42%). The maximum MP size in fish tissues ranged between 50–200 μm. For chemical characterization, 87 representative plastic-like particles were analyzed using Fourier transform infrared (FTIR) spectroscopy, revealing the presence of polychloroprene (18%), PA (15%), PEST (14%), polyacrylamide (13%), cellulose (10%), nylon (10%), polysulfide (7%), ethylene propylene (7%), and PTFE (6%) (Atamanalp et al., 2021). A study by Alfaro-Núñez et al. (2021), investigating MP contamination in the muscle tissue and digestive tract of 240 marine organisms from 16 species consumed by humans—including fish, cephalopod mollusks, and crustaceans—showed that 69% of the samples contained plastic fragments larger than 200 μm in the digestive tract. In the study, MPs were detected in 71% of 210 fish from 14 species (Alfaro-Núñez et al., 2021). Wootton et al. (2021) investigated the abundance of MPs in nine commercial fish species purchased from seafood markets across a 4000 km stretch of Australia, covering western Australia, South Australia, Victoria, Tasmania, and New South Wales. The study’s findings revealed that in all states, an average of 35.5% of fish samples had at least one piece of MP in the gastrointestinal tract (Wootton et al., 2021). Another study by Koongolla et al. (2022) investigated the MP abundance in 271 individuals of 32 species of marine fish collected from Beibu Bay. The results showed that the MP incidence rate was 93.7%. In the study, MP abundance ranged from 0.03 to 4 items, with a mean MP abundance of 1.02 ± 0.18 items. Characterization of MPs in fish species showed that fibers were the dominant shape (98%) and smaller MPs of 0.02–1 mm size were the dominant size (66%). Transparent (27%), black (25%), and blue (24%) were the predominant colors of MPs detected in fish, while the presence of red, green, yellow, and white MPs was also reported. In this study, PET (32%) and PP (21%) were the dominant polymers in MPs in fish collected from the onshore of Beibu Bay, while PVC (16%), polyacrylic (PAC) (11%), PEST (11%), poly methyl propenyl ether (PMPE) (5%), and polysulfide (5%) polymers were also detected in fish (Koongolla et al., 2022). In a study by Peters et al. (2017), analysis of 1381 fish from the Texas Gulf coast showed that approximately 42% of the fish had ingested MPs, with an average of 0.82 particles per fish. The study included 150 southern kingfish, 383 Atlantic croaker, 103 Atlantic spadefish, 139 sand trout, 449 pinfish, and 157 grunts. Of the total 1141 anthropogenic items detected, 1% were larger than 5 mm in size and 99% were smaller than 5 mm in size and included fiber, microbead, and fragment shapes. Fiber-shaped MPs accounted for more than 80% of the MPs ingested by southern kingfish (97.1%), pinfish (97.1%), sand trout (92.6%), and Atlantic croaker (83.5%), while they accounted for more than 60% for Atlantic spadefish (61.8%) and grunt (59.1%) (Peters et al., 2017).

As the size of MPs decreases, the potential for MPs to be retained in the gastrointestinal tract of fish increases (Critchell and Hoogenboom, 2018). MPs have negative effects on the body composition, growth rate, feed utilization rate, nutrient digestibility, swimming behavior, hematology, and histopathology of fish (Barboza et al., 2018; Mahmood et al., 2024; Rashid et al., 2024a; Rashid et al., 2024b). The swimming behavior of fish affects their ingestion of MPs, and fish with higher speed and acceleration may be more prone to ingest MPs (Li et al., 2024a) In relation to swimming behavior, MPs can cause a decrease in speed and resistance time during swimming, a decrease in range of motion, and hyperactive swimming behavior (Barboza et al., 2018; Yin et al., 2018; Chen et al., 2020). Furthermore, these negative effects of MPs on fish swimming behavior may impair their hunting ability. Moreover, MPs ingested by fish can lead to oxidative stress (Barboza et al., 2018), bioconcentration of metals in the gills (Barboza et al., 2018), bioaccumulation of metals in the liver (Barboza et al., 2018), thinning of the muscularis layer in the gut (Chen et al., 2020), gut microbiota dysbiosis (Zhang et al., 2021), liver damage (Zhang et al., 2021), disorders in lipid metabolism (Zhang et al., 2021), and the onset of obesity (Zhang et al., 2021).

Higher trophic level organisms such as sea turtles and seabirds may accidentally ingest plastics because they mistake the odor and visual stimuli of plastic for prey. Plastics may spontaneously contain odor chemicals and/or biofilms may form on plastics in the marine environment that emit dimethyl sulfide, an attractive chemical that acts as an olfactory sensory trap for many seabirds because it resembles the odor of their prey (Heswall et al., 2025). A recent study found that clear-white plastics are commonly found in the oceans globally and are commonly ingested by seabirds (Heswall et al., 2025). The researchers recommended minimizing the production and public use of clear-white plastics (Heswall et al., 2025). Although studies on MPs ingested by seabirds are very limited in the literature, a comprehensive study conducted by Navarro et al. (2023) in recent years reported that plastic debris was found in the gastrointestinal tract of 53 of 88 birds belonging to 14 species. The study found that plastics were present in the gastrointestinal tracts of 88.89% of Cory’s shearwaters (*Calonectris borealis*, n = 45), 100% of Madeiran storm-petrels (*Oceanodroma castro*, n = 5), 30% of yellow-legged gulls (*Larus michahellis*, n = 20), 50% of black-headed gulls (*Chroicocephalus ridibundus*, n = 2), and 100% of Cattle egrets (*Bubulcus ibis*, n = 1). In the study, 34% of the 371 items found in all sampled birds were analyzed using FTIR, showing that the most common plastic types were PE (70.87%) and PP (15.75%) (Navarro et al., 2023). It is worth noting that MP analyses conducted on seabird fecal samples provide a comprehensive perspective for evaluating MPs expelled from the organism following digestion, shedding light on potential biological elimination processes and helping to understand their possible roles as vectors. For instance, in a study conducted on fecal samples of Little auks (*Alle alle*), 25 potential MP particles (> 100 μm) were identified in 110 samples. The study reported an average of 0.08 ± 0.28 MP particles per fecal sample, with the detected MPs consisting of polymers such as PS, PP, and PET.

Throughout their life cycle, sea turtles’ active behavior and reliance on visual foraging strategies make them vulnerable to plastic pollution. Additionally, the presence of backward-facing esophageal papillae in sea turtles, which can facilitate the accumulation of particles in the intestines, may further increase their vulnerability to MP pollution. In a study analyzing the body tissues of ten loggerhead sea turtles (*Caretta caretta*)—including the kidneys, liver, spleen, heart, skeletal muscle, subcutaneous fat, stomach, intestines, and reproductive organs—microparticles were identified in 98.8% of all samples, approximately 70% of which were classified as MPs (Costello et al., 2025). The findings revealed that microparticles were most concentrated in the reproductive organs, followed by the heart, with PP and PE being the most common polymer types (Costello et al., 2025). Another recent study reported for the first time the presence of MPs—including polymers such as PE, PVC, and ABS—in the yolk and liver of sea turtle embryos (Chemello et al., 2023). While the effects of MPs on sea turtle health remain largely unknown, the detection of these pollutants in both adult reproductive tissues and embryonic organs raises concerns about their potential threat to reproductive health and the long-term sustainability of sea turtle populations, which are already facing multiple anthropogenic stressors during reproduction.

MP pollution also poses a significant threat to large marine species higher up the marine ecosystem food chain. Studies have shown the presence of MPs in the gastrointestinal tract of large marine species. One fin whale (*Balaenoptera physalus*), seven finless porpoises (*Neophocaena asiaeorientalis*), two loggerhead turtles (*Caretta caretta*), one Indo-Pacific bottlenose dolphin (*Tursiops aduncus*), and one common dolphin (*Delphinus delphis*) stranded off the coast of the Republic of Korea were investigated for MP abundance in the stomachs and intestines of stranded large marine animals, and the results showed that the mean MP abundance was 3.42 ± 3.2 items/g (Park et al., 2023). The dominant polymer type, color, shape, and size of MPs were PP, transparent-white, fragment, and < 200 μm, respectively (Park et al., 2023). In a study by Courville et al. (2024), 24 bottlenose dolphins (*Tursiops truncatus*), two pygmy sperm whales (*Kogia breviceps*), one pantropical spotted dolphin (*Stenella attenuata*), one short-snouted spinner dolphin (*Stenella clymene*), one Risso’s dolphin (*Grampus griseus*), and one dwarf sperm whale (*Kogia sima*) stranded in Alabama, Florida, Puerto Rico, and Texas revealed that there were 2871 microparticles in the gastrointestinal tracts of organisms, of which 12.5% were MPs. In the study, 10 polymer types were detected in the digestive tracts of odontocetes from the southeastern United States, including PET (37.4%), PE (22.1%), PA (11.7%), PP (9.5%), polyvinyl (7.8%), thermoplastic adhesive (4.2%), tire wear particles (4.2%), PS (2%), ACR (0.8%), and polyallomer. Fibers (59.5%) were the predominant form of MPs, followed by fragments (27.4%), films (9.8%), filaments (1.7%), and foams (1.7%). The study showed that white (50%) and black (27.1%) MPs predominated in the digestive tracts of odontocetes (Courville et al., 2024). A recent study by Liu et al. (2024) showed that pygmy blue whales (*Balaenoptera musculus brevicauda*) had an average of 0.82 items/g (dw) of MPs in fecal samples (n = 18). Researchers have reported that pygmy blue whales’ exposure to MP ingestion was as high as 1.74 × 10^6^ items/day and that MP ingestion is mainly due to trophic transfer (Liu et al., 2024). The presence of MPs in fecal samples indicates that organisms in marine ecosystems not only retain MPs in their gastrointestinal tracts but also excrete MPs through feces during their digestive processes.

## 5. Stakeholder-based solutions for ecosystem health

The “Zero Draft” of the international legally binding instrument on plastic pollution, including in the marine environment (UNEP/PP/INC.3/4), published by the United Nations (UN) on 4 September 2023, calls for cooperation among parties in the following areas: (1) identifying, assessing, and prioritizing accumulation zones, hotspots, and affected sectors; (2) taking effective mitigation and remediation measures for accumulation zones, hotspots, and sectors, taking into account the provisions of existing international agreements, including those related to the conservation and sustainable use of marine biodiversity; and (3) promoting public participation in environmentally sound remediation activities. Under the section on National Plans (Part IV.1) of the UNEP/PP/INC.3/4 document, a framework is provided for countries to develop their roadmaps to address plastic pollution. Each party is expected to develop and implement a national plan that includes key elements such as primary plastic polymers, problematic and avoidable plastic products, chemicals and polymers of concern, and strategies for reducing, reusing, refilling, and repairing plastics and plastic products, as well as measures to address plastic emissions and releases throughout their life cycle (UNEP, 2023). The implementation of such national plans and mitigation strategies requires active cooperation among stakeholders. Collaboration among stakeholders (manufacturers, consumers, researchers, and policymakers) in reducing plastic and MP pollution directly supports UN Sustainable Development Goal (SDG) 14: “Life below water” by contributing to the protection of marine ecosystems. In addition, decreasing the input of plastics into the marine environment helps minimize MP contamination in water sources, particularly in processes such as seawater desalination, thereby positively impacting UN SDG 6: “Clean water and sanitation”. Furthermore, it helps improve food security (UN SDG 2: “Zero hunger”) and protects human health (UN SDG 3: “Good health and well-being”) by reducing exposure to MPs in the food chain.

**Figure 2:**
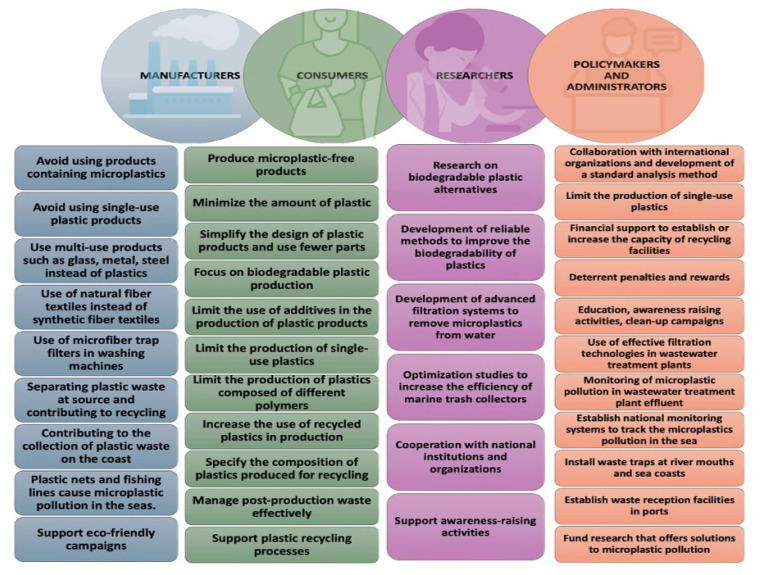
Responsibilities of stakeholders in reducing MP pollution in the marine environment.

Stakeholders have the responsibility to mitigate MP pollution in the marine environment (Figure 2). To reduce MP pollution in the environment, manufacturers should not use MPs in products and should prefer natural and eco-friendly alternatives. The US, UK, Canada, France, Sweden, China, and New Zealand are among the countries that have banned the use of microbeads through regulations or national laws (Habib et al., 2022). Since single-use plastic packaging contributes significantly to plastic waste, manufacturers should reduce its production to limit MP release into the marine environment. Minimizing the amount of plastics used in production, prioritizing the production of biodegradable plastics, using fewer parts in the design of plastic products, and limiting the production of new pellets by reintroducing recycled plastics into production processes contribute significantly to the role of manufacturers in reducing MP pollution. Single-use plastic bags, plates, cups, straws, cutlery, and packaging eventually end up in the oceans due to improper plastic waste management. Limiting the production of single-use plastics by plastic manufacturers helps reduce the amount of plastic waste, MP pollution, and pressure on plastic waste management.

Generally, recycled plastics are not preferred in the production process of a new product due to their higher cost compared to virgin plastics or their reduced quality (Shen and Worrell, 2024). Manufacturers may include a maximum percentage of recycled plastic content in their production processes due to marketing strategy, consumer demand, the achievement of sustainability goals, and legal obligations. Manufacturers should prioritize using the same type of polymers in plastic products to improve recyclability, as different polymers complicate the recycling process. Combining different plastic types and using additives to enhance product versatility reduces the recycling rate. Avoiding or limiting the use of pigments during plastic production makes recycling processes easier and helps obtain higher quality products in the recycling process. For instance, reprocessing different colors of 100% recyclable PET together leads to lower-quality recycled products (Singh and Walker, 2024).

While manufacturers play a crucial role, consumers also have significant responsibility for reducing plastic waste. The first step for consumers to contribute to minimizing marine plastic pollution may be to avoid using single-use plastics. Instead of plastic products, consumers should choose biodegradable and sustainable packaging materials such as reusable glass, metal, and steel. Refusing the use of toothpaste, shower gels, and peels containing MPs contributes to reducing the number of microbeads in wastewater treatment plants. Replacing synthetic textiles such as PEST, ACR, and nylon with natural fiber textiles such as cotton, linen, silk, and wool contributes significantly to the reduction of fiber-shaped MPs discharged from WWTPs into the marine environment. In addition, the use of washing machine filters helps capture significant amounts of microfibers, but the difficulty of installation, poor water drainage, and challenging filter cleaning are among the negative factors affecting the public adoption of washing machine filters (Abourich et al., 2024). Consumers can also prevent MPs from ending up in the marine environment by separating plastics at source in households and delivering them to plastic waste collection centers for effective recycling, participating in plastic waste collection events on beaches or collecting plastic waste from these areas individually, and prioritizing eco-friendly campaigns that reduce the amount of plastic waste in the environment and increase recycling.

**Figure 3:**
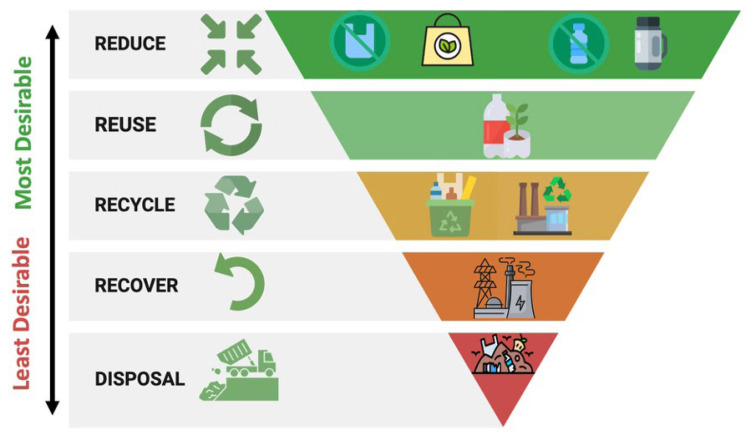
Waste hierarchy for plastic waste management.

The lack of a standardized method for MP analysis leads to the use of different pretreatments in studies, resulting in uncertainties in reported MP abundance. This prevents direct comparison of MP quantities in seawater and sediment samples between studies and hinders accurate assessment of the environmental distribution of MPs. An internationally standardized method of analysis could strengthen global collaborations by enabling more reliable determination of MP pollution. To reduce MP pollution in the seas, restricting the production of single-use plastic products at the national level, taxing consumers who use single-use plastics, banning the use of single-use plastics, and banning the production of products containing MPs contribute to reducing primary and secondary MP pollution in the environment. Policymakers and administrators should enforce regulations to minimize plastic pollution. In accordance with the 4R (reduce, reuse, recycle, recover) principle, the first step in an effective plastic waste management strategy is to reduce the amount of plastic waste, followed by reuse, recycling, and recovery (Figure 3).

Policymakers and administrators should develop and implement various strategies to ensure that landfilling is used only as a last resort in plastic waste management. Policymakers and administrators should encourage the establishment of recycling facilities to increase the recycling of plastic waste and provide financial support to expand the capacity of existing recycling facilities. Recycling facilities should use advanced filtration technologies to minimize the release of MPs during recycling, invest in advanced technologies that can recycle more types of plastics, and develop programs to ensure that plastic waste from more areas (e.g., households, industries, coastal areas, marine activities) is incorporated into the recycling system. The government or municipalities can offer reward-based incentives to consumers who separate their plastic waste at source and bring it to plastic waste collection centers for recycling, such as tax discounts and invoice deductions, based on the amount of plastic waste they bring. This would ensure that household plastic waste is effectively separated at source by consumers and that they play an active role in recycling processes. Conversely, strict monitoring of densely populated areas with camera surveillance systems and the imposition of high fines that can act as a deterrent to those who throw plastic and other waste into the environment may help minimize plastic pollution. Policymakers and practitioners should strengthen strategies for the effective collection and recycling of plastic waste, develop systems to monitor the abundance of MPs in the effluent of municipal wastewater treatment plants, strictly control MP pollution in industrial wastewater, ensure that treatment plants implement technologies that retain MPs with high efficiency, and conduct or support education and awareness-raising activities on plastic and MP pollution for the public, industries, and mariners.

Researchers and engineers should focus on developing effective treatment technologies or improving existing ones for the removal of MPs from large-scale municipal and industrial wastewater. Moreover, researchers should further study the advantages of different methods for MP analysis, biodegradable plastics, microorganisms that can biodegrade plastics, and optimization studies to improve the effectiveness of marine trash collectors.

## 6. Conclusion

The global entry of plastic and MPs from land-based and marine-based sources leads to increased accumulation of MPs in marine ecosystems. Although MP pollution in marine environments includes various polymer types, PE and PP are the most frequently detected due to their high global production volumes and low densities. MPs are not merely a standalone threat; rather, they represent a multifaceted risk. MPs affect organisms at all levels, from primary producers, phytoplankton, to large mammals. The effects of MPs on marine organisms vary depending on the abundance, characteristics, and duration of organisms’ exposure to MPs. The negative effects of MPs on cell growth, chlorophyll-a production, and photosynthetic activity in phytoplankton, which form the lowest level of the food web, lead to a decrease in primary production in the marine environment. Exposure of zooplankton to MPs decreases their survival, growth, reproduction, and feeding efficiency, and causes developmental abnormalities in their larvae. There are many negative effects on organisms at higher trophic levels, such as metabolic disorders, behavioral disorders, toxic accumulation in tissues, and organ damage. All these negative effects of MPs on marine organisms lead to the disruption of ecosystem functioning, the reduction of biodiversity, and the jeopardization of the sustainability of the marine ecosystem. Moreover, MPs of different polymer types contained in seafood, which plays an important role in the human diet, pose a potential risk to human health. MP pollution in marine environments fluctuates spatially and temporally depending on environmental conditions. To map global MP pollution and effectively address it, a standard MP analysis method needs to be developed for the sampling, extraction, and identification processes. The use of the standard MP analysis method on a global scale may allow the determination of priority areas to protect against MP pollution in marine environments. The solution to MP pollution requires an interdisciplinary approach that includes strong collaboration between manufacturers, consumers, policymakers, and researchers. The development of technologies that effectively remove MPs in wastewater treatment not only protects water quality but also reduces the risk of exposure of marine organisms to MPs. Investigating the potential use of biodegradable plastics helps prevent plastic waste from remaining in the marine environment for long periods. The use of microorganisms that can biodegrade MPs can support the improvement of marine ecosystems by contributing to natural bioremediation processes. Increasing studies on the development of inexpensive marine biodegradable plastics can pave the way for a sustainable way to prevent marine plastic pollution.

## Figures and Tables

**Table:** Abundance, shapes, colors, and polymer types of MPs in marine waters.

Location	Abundance	Shapes	Colors	Polymer types	Reference
Hamsilos Bay, Southern Black Sea	Range: 5.58–8.12 items/m^3^	Fiber, fragment, film, foam, other	Blue, transparent, red, black, white, grey, green, pink/purple, yellow, brown, mix	PET, PE PVC, PS	Öztekin et al. (2024)
Osaka Bay, Japan	Range: 8.9–22.8 items/L	Fragment, film, fiber, bead	Grey, colorless/white, black, red, yellow, blue, green, mixture	PP, PMMA, PEST, PE, PET, PVA, PVS, PU, PAA, polysilicate	Uaciquete et al.(2024)
Chukchi Sea, western Arctic Ocean	Range: 0–18,815 items/km^2^ Mean: 5236 ± 6127 items/km^2^	Fragment, pellet, line	-	PE, PP, PU, PET, PVC, PA, EVA, silicone resin	Ikenoue et al. (2023)
Izmir Bay (eastern Aegean Sea)	Mean: 1,083,882–8,091,684 items/km^2^	Fragment, fiber, film, foam, pellet	White, black, blue, transparent, red, green, pink, yellow, brown	PE, PP	Aksu et al. (2023)
Barents Sea	Range: 0.007–0.015 items/m^3^ Mean: 0.011 items/m^3^	Fiber, fragment	Blue, red, clear, clear/light blue, clear/light yellow, black	PEST, PA, PTFE, ACR, copolymer blends	Emberson-Marl et al. (2023)
Indian Ocean of the Southern Java Island	75–1013 items	Fragment, fiber, film, foam, pellet	Black, red, transparent, other	Tencel, bemberg, PTFE, FEP, Rayon, CP, POM, PB, PCTFE, PVC, PS, PP, PA, ABS.	Hakim et al. (2023)
Jakarta Bay, Indonesia	Mean: 55.8–86.6 items/L	Fiber, film, fragment and pellet	-	PE, PP	Takarina et al. (2022)
Sanya Bay, China	Range:15.50–22.14 items/L Mean: 18.37 ± 2.60 items/L	Fiber, fragment, film, fiber bundle	Black, blue, transparent,	PET, CP, PS, PP-PE, PP	Lei et al. (2021)
Eastern Mediterranean Sea	0.012–1.62 items/m^2^ Mean: 0.26 ± 0.36 items/m^2^	Fragment, filament, film, foam, pellet	-	PE, PP, PS, PA, PU, PVC, PVA, PA, PET, EPDM	Adamopoulou et al. (2021)
South China Sea	Range: 3000–19,000 items/m^3^ Mean: 8895 items/m^3^	Fragment, fiber, film and sphere	White, yellow, black, red, blue, and green	-	Zhang et al. (2020)
South Yellow Sea	Range: 4.5–6.5 items/L	Fiber, line, fragment, granule, film	Transparent, blue, black, red, yellow	PE, PP, PS, PA, PET, others	Jiang et al. (2020)
Western Mediterranean Sea, Italy	Mean: 69,161.3 ± 83,243.9 items/km^2^ Mean: 0.26 ± 0.33 items/m^3^	Fragment, sheet, filament, sphere	white, black, red, blue, transparent, green, other	PE, PP, PS, EVA, SBR	Baini et al. (2018)
Atlantic Ocean	Range: 0–8.5 items/m^3^ Mean: 1.15 ± 1.45 items/m^3^	Fiber, fragment	Blue, transparent, pink, other	PEST, blends, PA, PP, ACR, PVC, PS, PU	Kanhai et al. (2017)
Bohai Sea, China	Range: 0.01–1.23 items/m^3^ Mean: 0.33 ± 0.34 items/m^3^	Fragment, line, film, sphere, fiber, pellet, bead	White, transparent, green, yellow, black, blue, red	PE, PP, PS, PET, PU, PVC	Zhang et al. (2017)
Northeastern Pacific Ocean	8–9200 items/m^3^	Fiber/filament, angular and round fragments, films	Blue, red, black, purple	-	Desforges et al. (2014)

## References

[b1-tjb-49-05-421] Abourich M Bellamy A Ajji A Claveau-Mallet D 2024 Washing machine filters to mitigate microplastics release: Citizen science study to estimate microfibers capture potential and assess their social acceptability Environmental Challenges 16 100984 10.1016/j.envc.2024.100984

[b2-tjb-49-05-421] Acarer Arat S 2024a An overview of microplastic in marine waters: Sources, abundance, characteristics and negative effects on various marine organisms Desalination and Water Treatment 317 100138 10.1016/j.dwt.2024.100138

[b3-tjb-49-05-421] Acarer Arat S 2024b Microplastics in landfill leachate: Sources, abundance, characteristics, remediation approaches and future perspective Desalination and Water Treatment 319 100445 10.1016/j.dwt.2024.100445

[b4-tjb-49-05-421] Acarer S 2023a Abundance and characteristics of microplastics in drinking water treatment plants, distribution systems, water from refill kiosks, tap waters and bottled waters Science of The Total Environment 884 163866 10.1016/j.scitotenv.2023.163866 37142004

[b5-tjb-49-05-421] Acarer S 2023b Microplastics in wastewater treatment plants: Sources, properties, removal efficiency, removal mechanisms, and interactions with pollutants Water Science and Technology 87 3 685 710 10.2166/wst.2023.022 36789712

[b6-tjb-49-05-421] Adamopoulou A Zeri C Garaventa F Gambardella C Ioakeimidis C 2021 Distribution Patterns of Floating Microplastics in Open and Coastal Waters of the Eastern Mediterranean Sea (Ionian, Aegean, and Levantine Seas) Frontiers in Marine Science 8 699000 10.3389/fmars.2021.699000

[b7-tjb-49-05-421] Akdemir T Gedik K 2023 Microplastic emission trends in Turkish primary and secondary municipal wastewater treatment plant effluents discharged into the Sea of Marmara and Black Sea Environmental Research 231 116188 10.1016/j.envres.2023.116188 37230218

[b8-tjb-49-05-421] Aksu M Başaran A Sunlu U 2023 Spatio-temporal distribution of microplastic abundances in Izmir Bay (eastern Aegean Sea) Environmental Monitoring and Assessment 195 9 1116 10.1007/s10661-023-11790-w 37648952

[b9-tjb-49-05-421] Alfaro-Núñez A Astorga D Cáceres-Farías L Bastidas L Soto Villegas C 2021 Microplastic pollution in seawater and marine organisms across the Tropical Eastern Pacific and Galápagos Scientific Reports 11 1 6424 10.1038/s41598-021-85939-3 33742029 PMC7979831

[b10-tjb-49-05-421] Allen D Allen S Abbasi S Baker A Bergmann M 2022 Microplastics and nanoplastics in the marine-atmosphere environment Nature Reviews Earth & Environment 3 6 393 405 10.1038/s43017-022-00292-x

[b11-tjb-49-05-421] Aminah IS Ikejima K Vermeiren P 2023 Ingestion and translocation of microplastics in tissues of deposit-feeding crabs (Grapsoidea, Ocypodoidea) in Kochi estuary, Japan Marine Environmental Research 192 106252 10.1016/j.marenvres.2023.106252 37944348

[b12-tjb-49-05-421] Ammendolia J Covernton GA Skrepnyk A Dower JF Jacobs S 2025 Passing plastic: traces of plastic in the fecal samples of a high Arctic seabird in Tunu (East Greenland) Arctic Science 11 1 12 10.1139/as-2024-0014

[b13-tjb-49-05-421] Atamanalp M Köktürk M Uçar A Duyar HA Özdemir S 2021 Microplastics in Tissues (Brain, Gill, Muscle and Gastrointestinal) of Mullus barbatus and Alosa immaculata Archives of Environmental Contamination and Toxicology 81 3 460 469 10.1007/s00244-021-00885-5 34542666

[b14-tjb-49-05-421] Aydın İ Terzi Y Gündoğdu S Aytan Ü Öztürk RÇ 2023 Microplastic Pollution in Turkish Aquatic Ecosystems: Sources, Characteristics, Implications, and Mitigation Strategies Turkish Journal of Fisheries and Aquatic Sciences 23 12 TRJFAS24773 10.4194/TRJFAS24773

[b15-tjb-49-05-421] Baini M Fossi MC Galli M Caliani I Campani T 2018 Abundance and characterization of microplastics in the coastal waters of Tuscany (Italy): The application of the MSFD monitoring protocol in the Mediterranean Sea Marine Pollution Bulletin 133 543 552 10.1016/j.marpolbul.2018.06.016 30041348

[b16-tjb-49-05-421] Barboza LGA Vieira LR Guilhermino L 2018 Single and combined effects of microplastics and mercury on juveniles of the European seabass (Dicentrarchus labrax): Changes in behavioural responses and reduction of swimming velocity and resistance time Environmental Pollution 236 1014 1019 10.1016/j.envpol.2017.12.082 29449115

[b17-tjb-49-05-421] Botterell ZLR Beaumont N Cole M Hopkins FE Steinke M 2020 Bioavailability of Microplastics to Marine Zooplankton: Effect of Shape and Infochemicals Environmental Science & Technology 54 19 12024 12033 10.1021/acs.est.0c02715 32927944

[b18-tjb-49-05-421] Burghardt TE Pashkevich A Babić D Mosböck H Babić D 2022 Microplastics and road markings: the role of glass beads and loss estimation Transportation Research Part D: Transport and Environment 102 103123 10.1016/j.trd.2021.103123

[b19-tjb-49-05-421] Casabianca S Bellingeri A Capellacci S Sbrana A Russo T 2021 Ecological implications beyond the ecotoxicity of plastic debris on marine phytoplankton assemblage structure and functioning Environmental Pollution 290 118101 10.1016/j.envpol.2021.118101 34523510

[b20-tjb-49-05-421] Chemello G Trotta E Notarstefano V Papetti L Di Renzo L 2023 Microplastics evidence in yolk and liver of loggerhead sea turtles (Caretta caretta), a pilot study Environmental Pollution 337 122589 10.1016/j.envpol.2023.122589 37734631

[b21-tjb-49-05-421] Chen Q Lackmann C Wang W Seiler TB Hollert H 2020 Microplastics Lead to Hyperactive Swimming Behaviour in Adult Zebrafish Aquatic Toxicology 224 105521 10.1016/j.aquatox.2020.105521 32504859

[b22-tjb-49-05-421] Chen Q Shi G Revell LE Zhang J Zuo C 2023 Long-range atmospheric transport of microplastics across the southern hemisphere Nature Communications 14 1 7898 10.1038/s41467-023-43695-0 PMC10689495 38036501

[b23-tjb-49-05-421] Cole M Lindeque P Fileman E Halsband C Goodhead R 2013 Microplastic Ingestion by Zooplankton Environmental Science & Technology 47 12 6646 6655 10.1021/es400663f 23692270

[b24-tjb-49-05-421] Cole M Lindeque P Halsband C Galloway TS 2011 Microplastics as contaminants in the marine environment: A review Marine Pollution Bulletin 62 12 2588 2597 10.1016/j.marpolbul.2011.09.025 22001295

[b25-tjb-49-05-421] Corniuk RN Shaw KR McWhirter A Lynch HW Royer SJ 2023 Polymer identification of floating derelict fishing gear from O’ahu, Hawai’i Marine Pollution Bulletin 196 115570 10.1016/j.marpolbul.2023.115570 37776741

[b26-tjb-49-05-421] Costello L Zetterström A Gardner P Crespo-Picazo JL Bussy C 2025 Microplastics accumulate in all major organs of the mediterranean loggerhead sea turtle (Caretta caretta) Marine Environmental Research 208 107100 10.1016/j.marenvres.2025.107100 40203720

[b27-tjb-49-05-421] Courville JM Borkowski R Sonnenberg L Bielmyer-Fraser GK 2024 A Quantitative Analysis of Microplastics in the Gastrointestinal Tracts of Odontocetes in the Southeast Region of the United States Environmental Toxicology and Chemistry 43 6 1260 1273 10.1002/etc.5854 38546224

[b28-tjb-49-05-421] Cousins A Fenner N Aberg D Dunn C 2024 The Combined Effects of Ocean Warming and Microplastic Pollution on Marine Phytoplankton Community Dynamics 10.2139/ssrn.4947666 40513278

[b29-tjb-49-05-421] Critchell K Hoogenboom MO 2018 Effects of microplastic exposure on the body condition and behaviour of planktivorous reef fish (Acanthochromis polyacanthus) PLOS ONE 13 3 e0193308 10.1371/journal.pone.0193308 29494635 PMC5832226

[b30-tjb-49-05-421] d’Ambrières W 2019 Plastics recycling worldwide: current overview and desirable changes Field Actions Science Reports Special Issue 19 12 2021

[b31-tjb-49-05-421] Deng H Fu Q Li D Zhang Y He J 2021 Microplastic-associated biofilm in an intensive mariculture pond: Temporal dynamics of microbial communities, extracellular polymeric substances and impacts on microplastics properties Journal of Cleaner Production 319 128774 10.1016/j.jclepro.2021.128774

[b32-tjb-49-05-421] Desforges JPW Galbraith M Dangerfield N Ross PS 2014 Widespread distribution of microplastics in subsurface seawater in the NE Pacific Ocean Marine Pollution Bulletin 79 1–2 94 99 10.1016/j.marpolbul.2013.12.035 24398418

[b33-tjb-49-05-421] Desforges JPW Galbraith M Ross PS 2015 Ingestion of microplastics by zooplankton in the Northeast Pacific Ocean Archives of Environmental Contamination and Toxicology 69 3 320 330 10.1007/s00244-015-0172-5 26066061

[b34-tjb-49-05-421] Ding Y Zou X Wang C Feng Z Wang Y 2021 The abundance and characteristics of atmospheric microplastic deposition in the northwestern South China Sea in the fall Atmospheric Environment 253 118389 10.1016/j.atmosenv.2021.118389

[b35-tjb-49-05-421] Dipper F 2022 Open water lifestyles: marine plankton Elements of Marine Ecology Elsevier 193 228 10.1016/B978-0-08-102826-1.00005-3

[b36-tjb-49-05-421] Dong J Li L Liu Q Yang M Gao Z 2022 Interactive effects of polymethyl methacrylate (PMMA) microplastics and salinity variation on a marine diatom Phaeodactylum tricornutum Chemosphere 289 133240 10.1016/j.chemosphere.2021.133240 34896422

[b37-tjb-49-05-421] Emberson-Marl H Coppock RL Cole M Godley BJ Mimpriss N 2023 Microplastics in the Arctic: a transect through the Barents Sea Frontiers in Marine Science 10 124 1829 10.3389/fmars.2023.1241829

[b38-tjb-49-05-421] Eryaşar AR Mutlu T Karaoğlu K Veske E Gedik K 2024 Assessment of microplastic pollution in eleven commercial fish species in the Gulf of İzmir (Aegean Sea, eastern Mediterranean) Marine Pollution Bulletin 208 116932 10.1016/j.marpolbul.2024.116932 39270559

[b39-tjb-49-05-421] Fu L Li J Wang G Luan Y Dai W 2021 Adsorption behavior of organic pollutants on microplastics Ecotoxicology and Environmental Safety 217 112207 10.1016/j.ecoenv.2021.112207 33866287

[b40-tjb-49-05-421] Gamage S Mahagamage Y 2024 Microplastics in personal care products and cosmetics in Sri Lanka Heliyon 10 8 e29393 10.1016/j.heliyon.2024.e29393 38681610 PMC11053188

[b41-tjb-49-05-421] Gao L Fu D Zhao J Wu W Wang Z 2021 Microplastics aged in various environmental media exhibited strong sorption to heavy metals in seawater Marine Pollution Bulletin 169 112480 10.1016/j.marpolbul.2021.112480 34022557

[b42-tjb-49-05-421] Gao Y Fan KY Wei JX Zeng YY Liu QF 2023 Effects of microplastic concentrations on the survivability and reproduction of Moina macrocopa Applied Ecology and Environmental Research 21 5 4155 4163 10.15666/aeer/2105_41554163

[b43-tjb-49-05-421] Georgieva SK Peteva ZV Stancheva MD 2023 Evaluation of abundance of microplastics in the Bulgarian coastal waters BioRisk 20 59 69 10.3897/biorisk.20.97555

[b44-tjb-49-05-421] Gregory MR 2009 Environmental implications of plastic debris in marine settings—entanglement, ingestion, smothering, hangers-on, hitch-hiking and alien invasions Philosophical Transactions of the Royal Society B: Biological Sciences 364 1526 2013 2025 10.1098/rstb.2008.0265 PMC2873013 19528053

[b45-tjb-49-05-421] Guo Y Ma W Li J Liu W Qi P 2020 Effects of microplastics on growth, phenanthrene stress, and lipid accumulation in a diatom, Phaeodactylum tricornutum Environmental Pollution 257 113628 10.1016/j.envpol.2019.113628 31771928

[b46-tjb-49-05-421] Habib RZ Aldhanhani JAK Ali AH Ghebremedhin F Elkashlan M 2022 Trends of microplastic abundance in personal care products in the United Arab Emirates over the period of 3 years 2018–2020 Environmental Science and Pollution Research 29 59 89614 89624 10.1007/s11356-022-21773-y 35852742 PMC9294843

[b47-tjb-49-05-421] Hakim L Asmara AA Priambodo RY Wong YJ 2023 Microplastic pollution profile in the Indian Ocean of the Southern Java Island, Indonesia Environmental Challenges 13 100786 10.1016/j.envc.2023.100786

[b48-tjb-49-05-421] Haque MM Nupur FY Parvin F Tareq SM 2022 Occurrence and characteristics of microplastic in different types of industrial wastewater and sludge: A potential threat of emerging pollutants to the freshwater of Bangladesh Journal of Hazardous Materials Advances 8 100166 10.1016/j.hazadv.2022.100166

[b49-tjb-49-05-421] He P Chen L Shao L Zhang H Lü F 2019 Municipal solid waste (MSW) landfill: A source of microplastics? -Evidence of microplastics in landfill leachate Water Research 159 38 45 10.1016/j.watres.2019.04.060 31078750

[b50-tjb-49-05-421] Heswall AM Rayner M Wijaya BN Miller L Cain KE 2025 Clear-white plastics are most common in global oceans and seabird stomachs, but local species can ingest specific colours Marine Pollution Bulletin 215 117827 10.1016/j.marpolbul.2025.117827 40120356

[b51-tjb-49-05-421] Horie Y Mitsunaga K Yamaji K Hirokawa S Uaciquete D 2024 Variability in microplastic color preference and intake among selected marine and freshwater fish and crustaceans Discover Oceans 1 1 5 10.1007/s44289-024-00005-w

[b52-tjb-49-05-421] Hu C Xiao Y Jiang Q Wang M Xue T 2024 Adsorption properties and mechanism of Cu(II) on virgin and aged microplastics in the aquatic environment Environmental Science and Pollution Research 31 20 29434 29448 10.1007/s11356-024-33131-1 38575820

[b53-tjb-49-05-421] Ikenoue T Nakajima R Fujiwara A Onodera J Itoh M 2023 Horizontal distribution of surface microplastic concentrations and water-column microplastic inventories in the Chukchi Sea, western Arctic Ocean Science of The Total Environment 855 159564 10.1016/j.scitotenv.2022.159564 36332720

[b54-tjb-49-05-421] Isinibilir M Svetlichny L Mykitchak T Türkeri EE Eryalçın KM 2020 Microplastic consumption and its effect on respiration rate and motility of Calanus helgolandicus from the Marmara Sea Frontiers in Marine Science 7 603321 10.3389/fmars.2020.603321

[b55-tjb-49-05-421] Islam MS Islam ARMT Ismail Z Ahmed MK Ali MM Kabir MH Ibrahim KA Al-Qthanin RN Idris AM 2023 Effects of microplastic and heavy metals on coral reefs: A new window for analytical research Heliyon 9 11 e22692 10.1016/j.heliyon.2023.e22692 38074858 PMC10700871

[b56-tjb-49-05-421] Jang YL Jeong J Eo S Hong SH Shim WJ 2024 Occurrence and characteristics of microplastics in greywater from a research vessel Environmental Pollution 341 122941 10.1016/j.envpol.2023.122941 37979649

[b57-tjb-49-05-421] Jiang F Wang M Ding J Cao W Sun C 2022 Occurrence and seasonal variation of microplastics in the effluent from wastewater treatment plants in Qingdao China Journal of Marine Science and Engineering 10 1 58 10.3390/jmse10010058

[b58-tjb-49-05-421] Jiang Y Zhao Y Wang X Yang F Chen M 2020 Characterization of microplastics in the surface seawater of the South Yellow Sea as affected by season Science of The Total Environment 724 138375 10.1016/j.scitotenv.2020.138375 32408470

[b59-tjb-49-05-421] Kanhai LDK Officer R Lyashevska O Thompson RC O’Connor I 2017 Microplastic abundance, distribution and composition along a latitudinal gradient in the Atlantic Ocean Marine Pollution Bulletin 115 1–2 307 314 10.1016/j.marpolbul.2016.12.025 28007381

[b60-tjb-49-05-421] Kara N Sari Erkan H Onkal Engin G 2023 Characterization and removal of microplastics in landfill leachate treatment plants in Istanbul, Turkey Analytical Letters 56 9 1535 1548 10.1080/00032719.2022.2137808

[b61-tjb-49-05-421] Karadurmuş U Bilgili L 2024 Environmental impacts of synthetic fishing nets from manufacturing to disposal: A case study of Türkiye in life cycle perspective Marine Pollution Bulletin 198 115889 10.1016/j.marpolbul.2023.115889 38091633

[b62-tjb-49-05-421] Kole PJ Löhr AJ Van Belleghem F Ragas A 2017 Wear and tear of tyres: A stealthy source of microplastics in the environment International Journal of Environmental Research and Public Health 14 10 1265 10.3390/ijerph14101265 29053641 PMC5664766

[b63-tjb-49-05-421] Koongolla JB Lin L Yang CP Pan YF Li HX 2022 Microplastic prevalence in marine fish from onshore Beibu Gulf, South China Sea Frontiers in Marine Science 9 964461 10.3389/fmars.2022.964461

[b64-tjb-49-05-421] Kosore C Ojwang L Maghanga J Kamau J Kimeli A 2018 Occurrence and ingestion of microplastics by zooplankton in Kenya’s marine environment: first documented evidence African Journal of Marine Science 40 3 225 234 10.2989/1814232X.2018.1492969

[b65-tjb-49-05-421] Kvale K Prowe AEF Chien CT Landolfi A Oschlies A 2021 Zooplankton grazing of microplastic can accelerate global loss of ocean oxygen Nature Communications 12 1 2358 10.1038/s41467-021-22554-w PMC8060285 33883554

[b66-tjb-49-05-421] Le LT Nguyen KQN Nguyen PT Duong HC Bui XT 2022 Microfibers in laundry wastewater: Problem and solution Science of The Total Environment 852 158412 10.1016/j.scitotenv.2022.158412 36055511

[b67-tjb-49-05-421] Lee KW Shim WJ Kwon OY Kang JH 2013 Size-dependent effects of micro polystyrene particles in the marine copepod Tigriopus japonicus Environmental Science & Technology 47 19 11278 11283 10.1021/es401932b 23988225

[b68-tjb-49-05-421] Lei X Cheng H Luo Y Zhang Y Jiang L 2021 Abundance and characteristics of microplastics in seawater and corals from reef region of Sanya Bay, China Frontiers in Marine Science 8 728745 10.3389/fmars.2021.728745

[b69-tjb-49-05-421] Li B Liang W Fu S Fu C Cai Z 2024a Swimming behavior affects ingestion of microplastics by fish Aquatic Toxicology 266 106798 10.1016/j.aquatox.2023.106798 38104508

[b70-tjb-49-05-421] Li X Huang D Dong H Wen J Dong J 2024b Differential photoaging behaviors of different colored commercial polyethylene microplastics in water: The important role of color characteristics Science of the Total Environment 956 177361 10.1016/j.scitotenv.2024.177361 39500454

[b71-tjb-49-05-421] Li Y Neema P Andrews S 2024c Adsorption behavior and mechanisms of trihalomethanes onto virgin and weathered polyvinyl chloride microplastics Toxics 12 7 450 10.3390/toxics12070450 39058102 PMC11281136

[b72-tjb-49-05-421] Liu M Yang K Brannan N Liu HM Kudavidanage E 2024 Assessing exposure risk to microplastics by Indian Ocean pygmy blue whales Aquatic Toxicology 107166 10.1016/j.aquatox.2024.107166

[b73-tjb-49-05-421] Long Z Pan Z Wang W Ren J Yu X 2019 Microplastic abundance, characteristics, and removal in wastewater treatment plants in a coastal city of China Water Research 155 255 265 10.1016/j.watres.2019.02.028 30852313

[b74-tjb-49-05-421] Luu TT Truong DQ Nguyen VN Jeong S Nguyen 2025 Removal of microplastics from laundry wastewater using coagulation and membrane combination: A laboratory-scale study Membranes 15 2 47 10.3390/membranes15020047 39997673 PMC11857315

[b75-tjb-49-05-421] Mahdavi Soltani Z Cheraghi M Jaafarzadeh N Tavakkoli H 2022 Microplastics and microrubbers in soils around two landfills and a municipal solid waste transfer station in Ahvaz Metropolis, Iran Journal of Advances in Environmental Health Research 10 4 279 290 10.32598/JAEHR.10.4.1234

[b76-tjb-49-05-421] Mahesh S Gowda NK Mahesh S 2023 Identification of microplastics from urban informal solid waste landfill soil; MP associations with COD and chloride Water Science and Technology 87 1 115 129 10.2166/wst.2022.412 36640027

[b77-tjb-49-05-421] Mahmood M Hussain SM Sarker PK Ali S Arif MS 2024 Toxicological assessment of dietary exposure of polyethylene microplastics on growth, nutrient digestibility, carcass and gut histology of Nile Tilapia (Oreochromis niloticus) fingerlings Ecotoxicology 33 3 296 304 10.1007/s10646-024-02749-9 38498245

[b78-tjb-49-05-421] Mamtimin X Song W Wang Y Habibul N 2023 Arsenic adsorption by carboxylate and amino modified polystyrene micro- and nanoplastics: Kinetics and mechanisms Environmental Science and Pollution Research 30 15 44878 44892 10.1007/s11356-023-25475-x 36697988

[b79-tjb-49-05-421] Napper IE Wright LS Barrett AC Parker-Jurd FNF Thompson RC 2022 Potential microplastic release from the maritime industry: Abrasion of rope Science of the Total Environment 804 150155 10.1016/j.scitotenv.2021.150155 34520921

[b80-tjb-49-05-421] Navarro A Luzardo OP Gómez M Acosta-Dacal A Martínez I 2023 Microplastics ingestion and chemical pollutants in seabirds of Gran Canaria (Canary Islands, Spain) Marine Pollution Bulletin 186 114434 10.1016/j.marpolbul.2022.114434 36495613

[b81-tjb-49-05-421] Öztekin A Üstün F Bat L Tabak A 2024 Microplastic contamination of the seawater in the Hamsilos Bay of the Southern Black Sea Water, Air, & Soil Pollution 235 6 325 10.1007/s11270-024-07138-w

[b82-tjb-49-05-421] Park B Kim SK Joo S Kim JS Jo K 2023 Microplastics in large marine animals stranded in the Republic of Korea Marine Pollution Bulletin 189 114734 10.1016/j.marpolbul.2023.114734 36842279

[b83-tjb-49-05-421] Park B Yoon HJ Park H 2025 Development and efficiency evaluation of microplastic removal filter for laundry machines Water 17 3 358 10.3390/w17030358

[b84-tjb-49-05-421] Peters CA Thomas PA Rieper KB Bratton SP 2017 Foraging preferences influence microplastic ingestion by six marine fish species from the Texas Gulf Coast Marine Pollution Bulletin 124 1 82 88 10.1016/j.marpolbul.2017.06.080 28705629

[b85-tjb-49-05-421] Pfeiffer F Fischer EK 2020 Various digestion protocols within microplastic sample processing—Evaluating the resistance of different synthetic polymers and the efficiency of biogenic organic matter destruction Frontiers in Environmental Science 8 572424 10.3389/fenvs.2020.572424

[b86-tjb-49-05-421] Plastics Europe 2024 Plastics – the fast facts 2024

[b87-tjb-49-05-421] Pourebrahimi S Pirooz M 2023 Microplastic pollution in the marine environment: A review Journal of Hazardous Materials Advances 10 100327 10.1016/j.hazadv.2023.100327

[b88-tjb-49-05-421] Pratiwi OA Achmadi UF Kurniawan R 2024 Microplastic pollution in landfill soil: Emerging threats the environmental and public health Environmental Analysis Health and Toxicology 39 1 e2024009 10.5620/eaht.2024009 38631401 PMC11079410

[b89-tjb-49-05-421] Purwiyanto AIS Suteja Y Trisno Ningrum PS Putri WAE 2020 Concentration and adsorption of Pb and Cu in microplastics: Case study in aquatic environment Marine Pollution Bulletin 158 111380 10.1016/j.marpolbul.2020.111380 32568083

[b90-tjb-49-05-421] Rashid E Hussain SM Ali S Munir M Ghafoor A 2025 Impacts of microplastic accumulation in aquatic environment: Physiological, eco-toxicological, immunological, and neurotoxic effects Aquatic Toxicology 279 107232 10.1016/j.aquatox.2024.107232 39752783

[b91-tjb-49-05-421] Rashid E Hussain SM Ali S Sarker PK Al-Ghanim KA 2024a Microplastics accumulation in gut and revealing their impacts on nutritional quality and health of freshwater carp, Catla catla Aquaculture Reports 38 102299 10.1016/j.aqrep.2024.102299

[b92-tjb-49-05-421] Rashid E Hussain SM Sarker PK Ali S Paray BA 2024b Assessment of polystyrene microplastics as dietary additives in aquaculture species, Catla catla: Alters growth, feed utilization, nutritional composition, hematology and gut histopathology Aquaculture Reports 36 102100 10.1016/j.aqrep.2024.102100

[b93-tjb-49-05-421] Rummel CD Jahnke A Gorokhova E Kühnel D Schmitt-Jansen M 2017 Impacts of biofilm formation on the fate and potential effects of microplastic in the aquatic environment Environmental Science & Technology Letters 4 7 258 267 10.1021/acs.estlett.7b00164

[b94-tjb-49-05-421] Sharma VK Ma X Lichtfouse E Robert D 2023 Nanoplastics are potentially more dangerous than microplastics Environmental Chemistry Letters 21 4 1933 1936 10.1007/s10311-022-01539-1

[b95-tjb-49-05-421] Shen L Worrell E 2024 Plastic recycling Handbook of Recycling 497 510 10.1016/B978-0-323-85514-3.00014-2

[b96-tjb-49-05-421] Siddiqui S Dickens JM Cunningham BE Hutton SJ Pedersen EI 2022 Internalization, reduced growth, and behavioral effects following exposure to micro and nano tire particles in two estuarine indicator species Chemosphere 296 133934 10.1016/j.chemosphere.2022.133934 35176295 PMC9071364

[b97-tjb-49-05-421] Singh N Walker TR 2024 Plastic recycling: A panacea or environmental pollution problem Npj Materials Sustainability 2 1 17 10.1038/s44296-024-00024-w 39114578 PMC11304528

[b98-tjb-49-05-421] Soon ZY Tamburri MN Kim T Kim M 2024 Estimating total microplastic loads to the marine environment as a result of ship biofouling in-water cleaning Frontiers in Marine Science 11 10.3389/fmars.2024.1502000

[b99-tjb-49-05-421] Su Y Qi H Hou Y Gao M Li J 2022 Combined Effects of Microplastics and Benzo[a]pyrene on the Marine Diatom Chaetoceros muelleri Frontiers in Marine Science 8 779321 10.3389/fmars.2021.779321

[b100-tjb-49-05-421] Takarina ND Purwiyanto AIS Rasud AA Arifin AA Suteja Y 2022 Microplastic abundance and distribution in surface water and sediment collected from the coastal area Global Journal of Environmental Science and Management 8 2 183 196

[b101-tjb-49-05-421] Terzi Y Öztürk RÇ Eryaşar AR Yandi İ Şahin A 2025 Riverine microplastic discharge along the southern Black Sea coast of Türkiye Environmental Research Letters 20 2 024061 10.1088/1748-9326/adaf47

[b102-tjb-49-05-421] Thushari GGN Senevirathna JDM 2020 Plastic pollution in the marine environment Heliyon 6 8 e04709 10.1016/j.heliyon.2020.e04709 32923712 PMC7475234

[b103-tjb-49-05-421] Tsiota P Karkanorachaki K Syranidou E Franchini M Kalogerakis N 2018 Microbial Degradation of HDPE Secondary Microplastics: Preliminary Results Proceedings of the International Conference on Microplastic Pollution in the Mediterranean Sea 181 188 10.1007/978-3-319-71279-6_24

[b104-tjb-49-05-421] Uaciquete D Mitsunaga K Aoyama K Kitajima K Chiba T 2024 Microplastic abundance in the semi-enclosed Osaka Bay, Japan Environmental Science and Pollution Research 31 36 49455 49467 10.1007/s11356-024-34444-x 39078549

[b105-tjb-49-05-421] UNEP 2023 Zero draft text of the international legally binding instrument on plastic pollution, including in the marine environment UNEP/PP/INC.3/4

[b106-tjb-49-05-421] Van Do M Le TXT Vu ND Dang TT 2022 Distribution and occurrence of microplastics in wastewater treatment plants Environmental Technology & Innovation 26 102286 10.1016/j.eti.2022.102286

[b107-tjb-49-05-421] Vroom RJE Koelmans AA Besseling E Halsband C 2017 Aging of microplastics promotes their ingestion by marine zooplankton Environmental Pollution 231 987 996 10.1016/j.envpol.2017.08.088 28898955

[b108-tjb-49-05-421] Wang F Wang B Duan L Zhang Y Zhou Y 2020a Occurrence and distribution of microplastics in domestic, industrial, agricultural and aquacultural wastewater sources: A case study in Changzhou, China Water Research 182 115956 10.1016/j.watres.2020.115956 32622124

[b109-tjb-49-05-421] Wang L Yang H Guo M Wang Z Zheng X 2022 Adsorption of antibiotics on different microplastics (Mps) - behaviors and mechanisms SSRN Electronic Journal 10.2139/ssrn.4199145 36549518

[b110-tjb-49-05-421] Wang S Wang Y Liang Y Cao W Sun C 2020b The interactions between microplastic polyvinyl chloride and marine diatoms: Physiological, morphological, and growth effects Ecotoxicology and Environmental Safety 203 111000 10.1016/j.ecoenv.2020.111000 32736119

[b111-tjb-49-05-421] Wang T Li B Zou X Wang Y Li Y 2019 Emission of primary microplastics in mainland China: Invisible but not negligible Water Research 162 214 224 10.1016/j.watres.2019.06.042 31276985

[b112-tjb-49-05-421] Welden NA Cowie PR 2017 Degradation of common polymer ropes in a sublittoral marine environment Marine Pollution Bulletin 118 1–2 248 253 10.1016/j.marpolbul.2017.02.072 28267994

[b113-tjb-49-05-421] Wootton N Reis-Santos P Dowsett N Turnbull A Gillanders BM 2021 Low abundance of microplastics in commercially caught fish across southern Australia Environmental Pollution 290 118030 10.1016/j.envpol.2021.118030 34461419

[b114-tjb-49-05-421] Wright LS Napper IE Thompson RC 2021 Potential microplastic release from beached fishing gear in Great Britain’s region of highest fishing litter density Marine Pollution Bulletin 173 113115 34743074 10.1016/j.marpolbul.2021.113115

[b115-tjb-49-05-421] Xiong X Tu Y Chen X Jiang X Shi H 2019 Ingestion and egestion of polyethylene microplastics by goldfish (Carassius auratus): Influence of color and morphological features Heliyon 5 12 e03063 10.1016/j.heliyon.2019.e03063 32083206 PMC7019107

[b116-tjb-49-05-421] Yin L Chen B Xia B Shi X Qu K 2018 Polystyrene microplastics alter the behavior, energy reserve and nutritional composition of marine jacopever (Sebastes schlegelii) Journal of Hazardous Materials 360 97 105 10.1016/j.jhazmat.2018.07.110 30098534

[b117-tjb-49-05-421] Zhang C Wang S Sun D Pan Z Zhou A 2020 Microplastic pollution in surface water from east coastal areas of Guangdong, South China and preliminary study on microplastics biomonitoring using two marine fish Chemosphere 256 127202 10.1016/j.chemosphere.2020.127202 32470745

[b118-tjb-49-05-421] Zhang W Zhang S Wang J Wang Y Mu J 2017 Microplastic pollution in the surface waters of the Bohai Sea, China Environmental Pollution 231 541 548 10.1016/j.envpol.2017.08.058 28843202

[b119-tjb-49-05-421] Zhang X Wen K Ding D Liu J Lei Z 2021 Size-dependent adverse effects of microplastics on intestinal microbiota and metabolic homeostasis in the marine medaka (Oryzias melastigma) Environment International 151 106452 10.1016/j.envint.2021.106452 33639345

[b120-tjb-49-05-421] Zhao X Wang J Yee Leung KM Wu F 2022 Color: An important but overlooked factor for plastic photoaging and microplastic formation Environmental Science & Technology 56 13 9161 9163 10.1021/acs.est.2c02402 35729727

